# Isolated facial diplegia: A rare presentation of Guillain‐Barre syndrome

**DOI:** 10.1002/ccr3.4473

**Published:** 2021-07-23

**Authors:** Sundus Sardar, Sreethish Sasi, Suresh Menik Arachchige, Muhammad Zahid, Gayane Melikyan

**Affiliations:** ^1^ Department of Internal Medicine Hamad Medical Corporation Doha Qatar; ^2^ Department of Neurology Hamad Medical Corporation Doha Qatar

**Keywords:** Bell's palsy, bilateral facial palsy, facial diplegia, Guillain‐Barre syndrome

## Abstract

The paper presents a case of bilateral facial nerve palsy and its unique presentation. It discusses the etiologies of bilateral facial nerve palsy. We aim to provide awareness to its presentation, diagnosis, and management.

## Graphical abstract

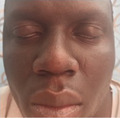

## 1 INTRODUCTION

Facial nerve palsy is a neurological condition that causes partial or complete impairment of the facial nerve. Bilateral facial nerve palsy is rare with an incidence of 1 per 5,000,000. We report the case of a 34‐year‐old gentleman who presented with sudden‐onset bilateral lower motor neuron (LMN) facial weakness.

Facial nerve palsy is a neurological condition in which the function of the facial nerve is partially or completely impaired. The most common presentation is unilateral, with an incidence of around 25 per 100,000 population, of which about 70% can be attributed to Bell's palsy.^1^ Lower motor neuron (LMN) facial diplegia represents a very small portion (about 2%) of all facial nerve palsy cases. Facial diplegia has an incidence of 1 per 5,000,000 population. The etiologies of this diplegia include Bell's palsy, Guillain‐Barré syndrome (GBS), idiopathic cranial neuropathies, Lyme disease, sarcoidosis, brainstem encephalitis, Miller Fisher syndrome, idiopathic intracranial hypertension, intracranial tumors, Syphilis, Hansen's disease, cryptococcal meningitis with acquired immunodeficiency syndrome and tuberculous meningitis.^2^ One study of 43 patients with bilateral facial nerve palsy showed that ten cases were attributed to Bell's palsy and five were due to GBS.^2^ There are several variants of GBS, and among these, facial diplegia with paresthesia is a rare variant. It is characterized by simultaneous facial diplegia, distal paresthesia, and minimal or no motor weakness. In defining facial diplegia, it is simultaneous if both sides are involved within 30 days of the initial onset of unilateral facial paralysis.^3^ We present a unique case of a young man with sudden‐onset isolated bilateral facial diplegia as an atypical presentation of Guillain‐Barré Syndrome.

## 2 CASE SUMMARY

**FIGURE 1:**
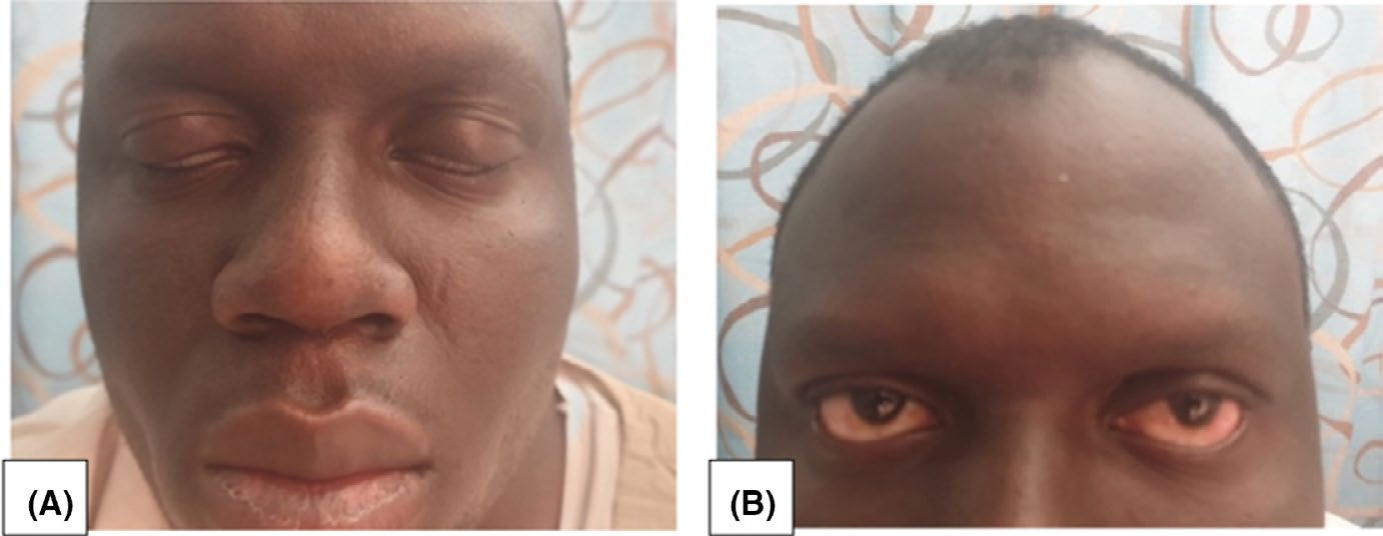
A, Incomplete closure of both eye‐lids (lagophthalmos). B, Inability to frown forehead

A 34‐year‐old Nigerian gentleman, with no significant past medical or surgical history, presented with one‐day history of sudden‐onset numbness and heaviness of the tongue. This was associated with drooling of saliva, loss of taste, slurring of speech, and the inability to close both eyes completely. The symptoms were persistent since onset and had never occurred before. He denied any earache, hearing difficulties, tinnitus, recent trauma to head or neck, or any other weakness or numbness in other parts of the body. There was no history of recent fever, flu‐like illness, nausea, vomiting, diarrhea, headache, neck stiffness, photophobia, alteration of the level of consciousness, or abnormal jerky body movements. He has been living in Qatar for the last 2 years and has never traveled to Europe or North America. There was no contact with sick persons, insect bites, or use of any herbal supplements. He has never smoked, used alcohol or any illicit drugs. Initial evaluation showed that he was afebrile with a temperature of 36.2℃ with heart rate of 81 beats per minute, the respiratory rate of 19 breaths per minute, a blood pressure of 117/69 mmHg, and oxygen saturation of 98% on room air. On general examination, he was not pale or icteric. On neurological examination, no visual field defects or loss in visual acuity was noted. The pupils were equal and reactive to light. There was full range of eye movements and no diplopia, but bilateral lagophthalmos was present (Figure 1A). Bell's phenomenon was also noted, where the eyes rolled upwards when the patient attempted to close his eyes. He was unable to clench his teeth, blow, whistle, or frown his forehead (Figure 1B). He was unable to show his teeth, and the nasolabial folds were inapparent bilaterally. Facial sensations and jaw strength were normal on both sides. Hearing, palatal tongue movements, head rotation, and shoulder shrug were intact. Ankle jerk was absent bilaterally, and all other deep tendon reflexes were diminished (1/4). Bulk, tone, motor strength, and sensation were normal in all four extremities. The remaining systemic examination was unremarkable.

Routine laboratory examinations and blood cultures were unremarkable (Table 1). Serum assays for anti‐neutrophil antibody (ANA), anti‐double‐stranded DNA antibody (anti‐dsDNA), and anti‐neutrophil cytoplasmic antibody (ANCA) were in the normal range. Polymerase chain reaction (PCR) examination of the blood for herpes and cytomegalovirus (CMV) viruses, viral capsid antigen for EBV, Quantiferon TB, serological tests of HIV, and Lyme disease were all negative. Analysis of cerebrospinal fluid showed albuminocytological dissociation with no evidence of infection (Table 2). A non‐contrast CT scan of the head showed no intra‐ or extracranial pathology. MRI brain with contrast and intracranial MRA were normal except for an incidental finding of a small nasopharyngeal retention/ Thornwaldt cyst. Chest X‐ray showed normal lung parenchyma, heart, and mediastinal silhouette. CT chest and whole‐body positron emission tomography (PET) CT scan did not reveal any evidence of underlying lymphoma, malignancy, sarcoidosis, or other paraneoplastic causes. Electromyography (EMG) and nerve conduction velocity (NCV) revealed a demyelinating type of facial palsy (Table 3). The blink reflex study revealed an abnormal bilateral blink reflex study which showed right‐sided absent ipsilateral R1 and R2, with absent contralateral R2, and absent ipsilateral and contralateral responses. He was started on intravenous immunoglobulin therapy at 0.4 g/kg for 5 days, along with ocular lubricants, eye patches, and physical therapy.

**TABLE 1:** Complete blood counts (CBC) and complete metabolic panel (CMP)

Test type	Value	Normal range
WBC (×10^3^/µl)	4.1	4–10
Neutrophil (%)	51.3	55–70
Lymphocyte (%)	35.8	20–40
Monocyte (%)	11.7	2–8
Eosinophil (%)	0.5	1–4
Basophil (%)	0.7	0.5–1
Hb (gm/dl)	15.1	12–15
Platelets (×10^3^/µl)	306	150–400
Urea (mmol/L)	2.4	2.5–6.7
Cr (µmol/L)	87	50–98
Na (mmol/L)	141	136–145
K (mmol/L)	3.9	3.5–5.1
Cl (mmol/L)	103	98–107
Bicarbonate (mmol/L)	26	22–29
Calcium (mmol/L)	2.43	2.15–2.50
Glucose (mmol/L)	5.2	3.3–5.5
CRP (mg/L)	1.8	0–5.0

Abbreviations: µl, microliters; gm/dl, grams/deciliter; mmol/L, millimoles per liter; µmol/L, micromole per liter; mg/L, milligrams per liter.

**TABLE 2:** Results of cerebro spinal fluid analysis

Detail	Value w/Units	Normal range
Color of CSF	Colorless	
Appearance CSF	Clear	
WBC CSF (/µl)	1	0–5
RBC CSF (/µl)	1	0–2
CSF Glucose (mmol/L)	3.25	2.22–3.89
CSF Protein (gm/L)	0.75	0.15–0.45
CSF LDH (U/L)	15.0	<40
CSF Culture	Negative	
CSF AFB Smear, PCR, and Culture	Negative	

Abbreviations: µl, microliters; mmol/L, millimoles per liter; gm/L, grams per liter; U/L, International units per liter.

**TABLE 3:** Nerve conduction studies

Motor nerves	Lat SD (ms)	Amp SD (mV)	CV SD (m/s)	Amp% SD (%)	F‐M SD (ms)
Right median					
Pos. 1–Rec pos	6.2	15.4			28.4
Pos. 2–Pos. 1	11.6	14.1	50.0	−8	
Left median					
Pos. 1–Rec pos	6.8	20.8			31.6
Pos. 2–Pos. 1	12.0	16.3	51.9	−22	
Right Ulnar					
Pos. 1–Rec pos	3.2	18.6			32.8
Pos. 2–Pos. 1	8.6	17.0	50.0	−9	
Pos. 3–Pos. 2	10.4	16.7	55.6	−2	
Left Ulnar					
Pos. 1–Rec pos	3.2	19.0			31.8
Pos. 2–Pos. 1	8.5	18.6	47.2	−2	
Pos. 3–Pos. 2	9.9	18.7	64.3	1	
Right Tibial					
Pos. 1–Rec pos	6.3	10.2			60
Pos. 2–Pos. 1	16.8	10.6	40	5	
Left Tibial					
Pos. 1–Rec pos	5.5	8.2			56.2
Pos. 2–Pos. 1	16.3	8.3	41.7	1	
Right Peroneal					
Pos. 1–Rec pos	6	6.8			53.3
Pos. 2–Pos. 1	14.6	5.6	40.7	−18	
Pos. 3–Pos. 2	15.9	5.0	61.5	−11	
Right Facial r.aur.					
Pos. 1–Rec pos	2.5	4.5			
Right Facial r.aur.					
Pos. 1–Rec pos	2.2	4.5			

**FIGURE 2:**
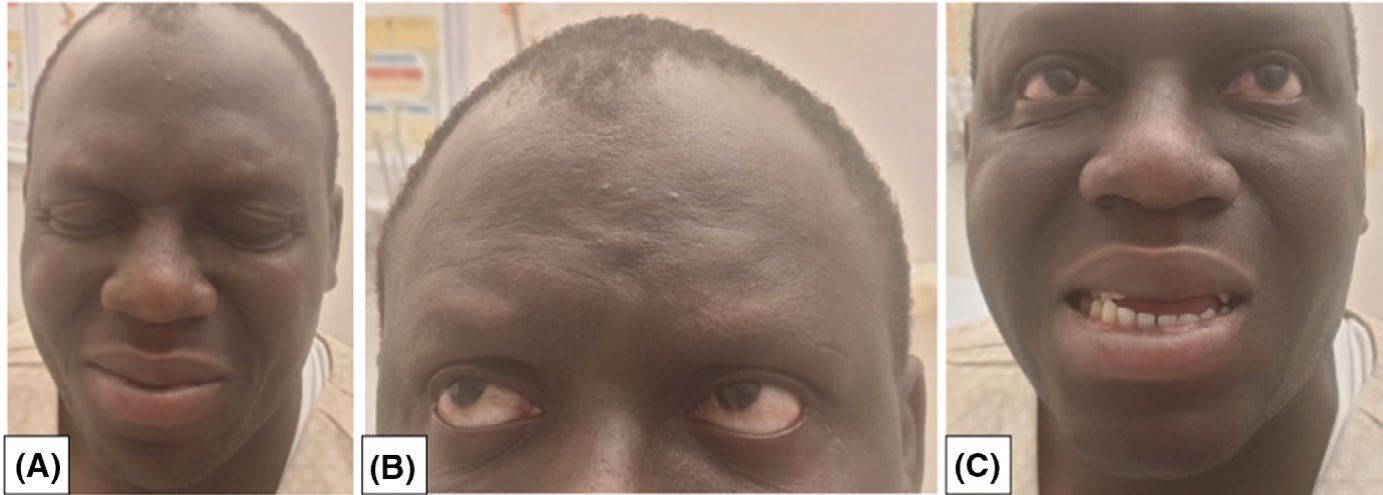
A, Able to completely close both eyes. B, Able to frown his forehead. C, Inapparent right nasolabial fold with mild deviation of the right side of the mouth

Follow‐up at one month showed significant improvement with complete resolution and thus ability to close his eyes (Figure 2A) and frown his forehead (Figure 2B). However, he still had inapparent right nasolabial fold with mild deviation of the right side of the mouth (Figure 2C). Follow‐up nerve conduction velocity at two‐month interval revealed unremarkable blink study with normal latencies and CAMP amplitudes of ipsilateral R1, R2, and contralateral R2.

## 3 DISCUSSION

Most cases of unilateral LMN facial nerve palsy are idiopathic, referred to as Bell's palsy. However, cases of bilateral LMN facial nerve palsy are predominantly attributed to underlying systemic conditions.^3^ GBS is an autoimmune‐mediated polyradiculoneuropathy characterized by rapidly evolving ascending weakness, mild sensory loss, and hypo‐ or areflexia, progressing to a nadir over up to 4 weeks. Evaluation of cerebrospinal fluid demonstrates albuminocytologic dissociation in 90% of cases. Acute inflammatory demyelinating polyneuropathy (AIDP) is the most common form of GBS in which the immune attack is directed at peripheral nerve myelin. Besides this classic presentation, there are clinical variants based on the types of nerve fibers involved (motor, sensory, sensory and motor, cranial or autonomic), the predominant mode of fiber injury (demyelinating versus axonal), and the presence of alteration in consciousness.^4^ Acute motor axonal neuropathy (AMAN) is unique as there is no sensory involvement, and the reflexes are preserved. Acute motor and sensory axonal neuropathy (AMSAN), Miller Fisher syndrome (MFS), Bickerstaff encephalitis, pharyngeal‐cervical‐brachial weakness, pan‐dysautonomia, pure sensory GBS, sixth nerve palsy with paresthesia, and facial diplegia with paresthesia (FDP) are the other rare variants of GBS.^5^ Isolated facial diplegia is a rare variant of GBS, with only a few cases described so far. Deep tendon reflexes are generally absent but rarely can be present or even exaggerated.^6^ As in the case of our patient, ankle reflexes were absent, and knee and upper limb reflexes were decreased. Diagnosis of GBS is based on clinical examination, CSF analysis, and neurophysiological studies. CSF shows albuminocytological dissociation, as seen in our case. There have been cases where IgG antibodies to galactocerebroside and phosphatidic acid are evident, indicating their possible use as a diagnostic marker.^7^, ^8^, ^9^, ^10^, ^11^ However, in our case, these antibodies were not sent. NCV and EMG are helpful in establishing a diagnosis of GBS;^7^, ^8^ however, may often have nonspecific EMG findings in patients with facial diplegia. Studies have showed that NCV, in facial diplegia subtype of GBS, is consistent with a demyelinating picture with an abnormal blink reflex.^9^, ^10^ In our patient, the results show an abnormal blink reflex which had improved at follow‐up. Furthermore, the NCV is consistent with a demyelinating picture which is in line with evidence in the literature.

Treatment of GBS includes supportive care and disease‐modifying therapy with intravenous immunoglobulin (IVIG) or plasma exchange (PE).^8^, ^9^, ^10^ Commonly, bilateral FN palsy will initially start from one side and progress to involve the other side subsequently. Our case is rare due to the synchronous presentation of bilateral facial involvement on the initial presentation. Approximately 80% of patients diagnosed with GBS are able to independently walk at six months with complete recovery of motor strength in 60% of patients at 1 year.^9^, ^10^, ^11^ Up to 10% of patients with GBS may experience relapses with increased weakness. In conclusion, our case was unique as the onset of facial palsy was sudden on both sides and not associated with any distal paresthesia and later progressed to absent ankle reflexes and decreased knee and upper extremity reflexes. Absence of deep tendon reflexes, albuminocytological dissociation of CSF, NCV consistent with the literature, and improvement with IVIG favored a diagnosis of GBS variant, while all other possible differentials were ruled out through appropriate tests.

## 4 ACKNOWLEDGEMENTS

Published with written consent of the patient.

## DATA AVAILABILITY STATEMENT

The authors declare that the data supporting the findings of this case report are available within the article. The corresponding author can be contacted for further information or clarification.

## CONFLICT OF INTEREST

There authors involved in this paper have no conflict of interest to declare.

## AUTHOR CONTRIBUTIONS

Drs. Suresh Menik Arachchige, Sundus Sardar, and Sreethish Sasi were equally involved with the literature search, writing, and editing the paper. Drs. Muhammad Zahid and Gayane Melikyan were involved with editing the paper and proving guidance.

## ETHICAL STATEMENT

This paper is the authors’ original work and has not be previously published or awaiting publication on another journal.
